# Low-dose radiation therapy suppresses viral pneumonia by enhancing broad-spectrum anti-inflammatory responses via transforming growth factor-β production

**DOI:** 10.3389/fimmu.2023.1182927

**Published:** 2023-05-25

**Authors:** Ha-Yeon Song, Fengjia Chen, Hae Ran Park, Jeong Moo Han, Hyun Jung Ji, Eui-Baek Byun, Yeongkag Kwon, Min-Kyu Kim, Ki Bum Ahn, Ho Seong Seo

**Affiliations:** ^1^ Research Division for Radiation Science, Advanced Radiation Technology Institute, Korea Atomic Energy Research Institute, Jeongeup, Republic of Korea; ^2^ Department of Biotechnology, College of Life Science and Biotechnology, Korea University, Seoul, Republic of Korea; ^3^ Department of Oral Microbiology and Immunology, Dental Research Institute (DRI), and BK21 Plus Program, School of Dentistry, Seoul National University, Seoul, Republic of Korea; ^4^ Animal Production and Health Laboratory, Joint Food and Agricultural Organization/International Atomic Energy Agency (FAO/IAEA) Centre for Nuclear Applications in Food and Agriculture, Department of Nuclear Sciences and Applications, International Atomic Energy Agency, Seibersdorf, Austria; ^5^ Department of Radiation Science, University of Science and Technology, Daejeon, Republic of Korea

**Keywords:** low-dose radiation therapy, LDRT, viral pneumonia, cytokine storm, transforming growth factor-β, anti-inflammatory therapy

## Abstract

Low-dose radiation therapy (LDRT) can suppress intractable inflammation, such as that in rheumatoid arthritis, and is used for treating more than 10,000 rheumatoid arthritis patients annually in Europe. Several recent clinical trials have reported that LDRT can effectively reduce the severity of coronavirus disease (COVID-19) and other cases of viral pneumonia. However, the therapeutic mechanism of LDRT remains unelucidated. Therefore, in the current study, we aimed to investigate the molecular mechanism underlying immunological alterations in influenza pneumonia after LDRT. Mice were irradiated to the whole lung 1 day post-infection. The changes in levels of inflammatory mediators (cytokines and chemokines) and immune cell populations in the bronchoalveolar lavage (BALF), lungs, and serum were examined. LDRT-treated mice displayed markedly increased survival rates and reduced lung edema and airway and vascular inflammation in the lung; however, the viral titers in the lungs were unaffected. Levels of primary inflammatory cytokines were reduced after LDRT, and transforming growth factor-β (TGF-β) levels increased significantly on day 1 following LDRT. Levels of chemokines increased from day 3 following LDRT. Additionally, M2 macrophage polarization or recruitment was increased following LDRT. We found that LDRT-induced TGF-β reduced the levels of cytokines and polarized M2 cells and blocked immune cell infiltration, including neutrophils, in BALF. LDRT-induced early TGF-β production was shown to be a key regulator involved in broad-spectrum anti-inflammatory activity in virus-infected lungs. Therefore, LDRT or TGF-β may be an alternative therapy for viral pneumonia.

## 1 Introduction

Coronavirus disease (COVID-19), caused by severe acute respiratory syndrome coronavirus 2 (SARS-CoV-2), previously unknown to humans, is spreading rapidly spreading across the globe, collapsing national economic, medical, social, and/or political systems that are unprepared to address the pandemic risk (1, 2). The current clinical understanding of the pathophysiological progression of COVID-19 is divided into three phases: (i) early-infection phase with mild or no symptoms of upper respiratory tract infection; (ii) pulmonary phase when the patients develop pneumonia-relevant symptoms (hypoxemia, tachypnea, shortness of breath); (iii) hyper-inflammation phase when the patients develop acute respiratory distress syndrome (ARDS), shock, and multi-organ failure. It has been reported that 67% of COVID-19 patients with a fatal illness have developed ARDS, a life-threatening condition that develops when the lungs become severely inflamed due to pneumonia, influenza, or injury (3). The excessive inflammatory response caused by COVID-19 ARDS in the lungs, referred to as a cytokine storm (CS), is considered to be linked with SARS-CoV-2 mortality (4).

Low-dose radiation therapy (LDRT), historically used for non-resolving pneumonitis during the 20^th^ century, has been recently considered an effective broad-spectrum approach for blocking CS caused by SARS-CoV-2 and other cases of viral pneumonia (5, 6). After the advent of the COVID-19 pandemic, Dr. Clayton Hess of Emory University used this old strategy and conducted a clinical trial (NCT04366791) on five patients with severe COVID-19. Four patients recovered rapidly without acute toxicity (7, 8). Similar findings from Spain showed that patients showed improved respiratory parameters and biochemical results after radiotherapy at a single dose of 0.5 Gy (9). Several clinical trials are underway to examine the efficacy of LDRT against COVID-19 (8, 10). Many researchers speculate that the effectiveness of LDRT on COVID-19 pneumonia is primarily because of its anti-inflammatory effects. However, there are doubts about its safety and efficacy because of limited preclinical information and a need for more effective controls in clinical trials (11).

Despite limited studies regarding the detailed radiobiological mechanism of LDRT compared to that of high-dose RT for cancer, several immunological response mechanisms have been proposed (12). One mechanism is that LDRT may induce an anti-inflammatory response, leading to M2 macrophage polarization (13, 14). LDRT may also inhibit the interaction between leukocytes and endothelial cells by downregulating the levels of chemokines and pro-inflammatory cytokines (15, 16). Meziani et al. demonstrated that IL-10 produced by neuronal and airway-associated macrophages after LDRT plays a vital role in the anti-inflammatory effect of influenza-induced pneumonia (17). Nevertheless, studies on the basic molecular mechanisms of immune suppression for the inhibition of pneumonia by LDRT are limited. Therefore, a comprehensive analysis and preclinical study of the immunological alterations induced by LDRT to treat CS are required. We, therefore, aimed to analyze the molecular mechanisms underlying LDRT-induced immunological changes and alleviation of pneumonia in an influenza virus-induced severe pneumonia mouse model.

## 2 Materials and methods

### 2.1 Ethical approval

All protocols of animal experiments were approved by the Committee on the Use and Care of Animals of KAERI (No. KAERI-IACUC-2021-003). All procedures were performed according to the KAERI Animal Care Center’s accepted veterinary standards.

### 2.2 Antibodies and reagents

Purified rat anti-mouse CD16/CD32 (#553142), V450-conjugated rat anti-mouse CD45 (#560501), BV510-conjugated rat anti-mouse Ly6G (#740157), FITC-conjugated rat anti-mouse CD11b (#557396), FITC-conjugated rat anti-mouse CD86 (#561962), Alexa Fluor 647-conjugated mouse anti-mouse CD64 (#558539), Alexa Fluor 647-conjugated anti-mouse CD206 (#565250), PE-conjugated hamster anti-mouse CD11c (#553802), and PE-CF594-conjugated anti-mouse CD11c (#562454) were purchased from BD Biosciences (San Diego, CA, USA). In addition, PE-Texas Red-conjugated anti-mouse CD11b (#RM2817) and PE-Cy7-conjugated rat anti-mouse MHC-II (#25-5321-82) were purchased from Thermo Fisher Scientific (Waltham, MA, USA). PE-conjugated mouse anti-mouse CD64 (#139304) was purchased from BioLegend (San Diego, CA, USA). Anti-mouse/human/rat/monkey/hamster/canine/bovine TGF-β (#BE0057) and mouse IgG1 isotype control (#BE0083) antibodies were purchased from BioXCell (Lebanon, NH, USA). Unless indicated otherwise, all chemicals used in this study were purchased from Sigma-Aldrich (#SML1633; St. Louis, MO, USA).

### 2.3 Mouse experiments

Specific pathogen-free, female, 6–7-week-old BALB/c mice were purchased from Orient Bio Inc. (Seoul, Korea). The animal housing conditions, designed for specific pathogen-free animals, were approved by the Committee on the Use and Care of Animals at the KAERI. The individually ventilated cages (Orient Bio Inc.) were maintained in an animal biological safety level (BSL) 2 facility at 22–23°C under a 12-h:12-h light: dark cycle.

Influenza A/Solomon Islands/03/06 (H1N1) virus was obtained from Korea University Guro Hospital (Seoul, Korea). The viruses were propagated and harvested from the allantoic cavities of 10-day-old SPF embryonated chicken eggs (18). Mice were intranasally infected with 30 hemagglutination units (HAU) of influenza A virus in 50 μL of sterile PBS under anesthesia using 2,2,2-tribromoethanol. Mice in the normal group were administered PBS alone as the control. All experiments using live viruses or animals were carried out in BSL2 containment laboratories, as approved by the KAERI Biosafety Committee.

At the indicated time points, the mice were euthanized using CO_2_ asphyxiation, and blood was collected using a capillary tube from the medial canthus of the eye. BALF was obtained by flushing the lungs twice with 0.5 mL/time of cold PBS. BALF cells were isolated by centrifugation at 400 × *g* for 5 min at 4°C. The BALF supernatant was stored at -80 °C until required. Virus titration in BALF was performed using a 50% tissue culture infectious dose (TCID_50_) assay using MDCK cells as described in a previously established method (19).

TGF-β1 neutralization was performed as described previously (20). Mice were intraperitoneally injected with monoclonal anti-TGF-β antibody (200 μg/mouse, #BE0057; BioXCell) or mouse IgG1 isotype control (#BE0083, BioXCell) 4 h before thoracic irradiation.

### 2.4 Irradiation experiment

After 24 h of influenza A virus infection, the mice were immobilized by anesthesia (ketamine 100 mg/kg and xylazine 10 mg/kg) and locally irradiated at doses of 0.6 Gy and 1.8 Gy on the thorax using a radioactive ^137^Cs gamma irradiator (Gamma cell-40; Nordin International, Inc., Ottawa, Canada). The dose rate was 0.47 Gy/min. A lead shield (diameter: 3.5 cm, thickness: 2.5 cm) was used to prevent irradiation outside the thorax. Body weight loss was monitored daily until day 12 post-irradiation. Mice with a weight loss of >20% of their starting weight were euthanized and recorded as dead.

### 2.5 Lung histopathology

The left lung lobes were fixed with 10% formalin for histopathological examination. Lung tissues were dehydrated, gradually soaked in alcohol and xylene, and embedded in paraffin. Tissue sections (5 μm) were deparaffinized and stained with H&E (#ab245880; Abcam, Cambridge, UK). The stained sections were digitally scanned using the Motic EasyScan slide scanning system. Histopathological evaluation was performed in a blinded manner by determining airway and vascular inflammation (21).

### 2.6 Quantification of serum C-reactive protein levels

Serum was obtained after centrifugation of the blood samples. Serum CRP levels were measured using an automatic biochemical analyzer (DRI-CHEM 4000i; Fujifilm; Tokyo, Japan).

### 2.7 Analysis of cytokines and chemokines in BALF

The levels of TNF-α (#558299), IL-6 (#558301), IL-1β (#560232), IL-10 (#558300), IFN-γ (#558296), IL-2 (#558297), IL-12p70 (#558303), RANTES (#558345), MCP-1 (#558342), KC (#558340), and MIP-1α (#558449) in BALF were measured using a cytometric bead array kit (BD Biosciences). Microbeads with different intensities coated with capture antibodies specific for each cytokine and chemokine were added and mixed with 50 μL BALF for 1 h in the dark. Following incubation, the phycoerythrin (PE) detection reagent was added and incubated for 1 h. The mixtures were washed and analyzed using a flow cytometer (MACSQuant VYB; Miltenyi Biotec, Bergish Gladbach, Germany). The active TGF-β in the BALF was measured using a commercial ELISA kit (#88-8350-77, Thermo Fisher Scientific) according to the manufacturer’s protocol without sample acidification.

### 2.8 Analysis of immune cells in BALF

Immune cells in BALF were analyzed following a previously established protocol with slight modification (22). Single-cell suspensions obtained from BALF were incubated with anti-mouse CD16/CD32 (Mouse BD Fc Block™) and then stained with V450-conjugated anti-mouse CD45, BV510-conjugated anti-mouse Ly6G, FITC-conjugated anti-CD11b, PE-conjugated anti-CD11c, PE-Cy7-conjugated anti-MHC-II, PE-CF594-conjugated anti-CD24, Alexa Fluor 647-conjugated anti-CD64, and 7-aminoactinomycin D (7-AAD) solution for 30 min at 4°C. After staining, the cells were washed and the absolute number of leukocytes was counted using flow cytometry.

To determine the phenotypic changes in macrophages, the cells were stained with V450-conjugated anti-mouse CD45, PE-conjugated anti-CD64, PE-CF594-conjugated anti-mouse CD11c, PE-Texas Red-conjugated anti-CD11b, anti-CD11c, Alexa Fluor 647-conjugated anti-CD206, FITC-conjugated anti-CD86, PE-Cy7-conjugated anti-MHC-II, and the LIVE/DEAD™ Fixable Aqua Dead Cell Stain Kit for 30 min at 4°C. In addition, the expression level of CD86 and CD206 cells were gated on CD45^+^CD11b^+^CD11c^+^MHC-II^+^CD64^+^ cells as a total macrophage in BALF. Data were collected using flow cytometry and analyzed using FlowJo version 10 (TreeStar, Ashland, OR, USA).

### 2.9 Analysis of blood leukocytes

Whole blood was collected from the retro-orbital veins using heparin-coated capillary tubes in K_3_EDTA tubes. The absolute number of white blood cells, monocytes, and neutrophils in the blood was counted using an automatic blood analyzer (Hemavet 950; CDC Technologies Inc., Dayton, OH, USA).

### 2.10 Quantitative real-time polymerase chain reaction

The mouse lungs were collected three days after irradiation, and the total RNA was isolated using the TRIzol Reagent (Thermo Fisher Scientific) according to the manufacturer’s protocol. Reverse transcription on 3 µg of total RNA was performed using random primers, dNTP mixture, and MMLV reverse transcriptase (Promega, Madison, WI, USA). Quantitative real-time PCR was performed using StepOne Real-Time PCR (Applied Biosystems, Foster City, CA, USA) with the SYBR Green reagent (Takara, Tokyo, Japan). Primers were designed using Primer-BLAST, and the sequences are presented in **Supplementary Table 1**. The comparative Ct method was used, and relative mRNA expression was calculated based on normalizing the β-actin expression. All experiments were repeated thrice.

### 2.11 Analysis of lung fibrosis

SPF, female, 6-week-old C57BL/6 mice were locally irradiated at 1.8 Gy and 10 Gy (positive control for lung fibrosis) to the thorax using a Gamma cell-40. Histological lung inflammation and fibrosis scores were determined using the H&E-stained or Masson’s trichrome (MT)-stained sections.

The level of TGF-β1 in lung homogenates was measured using a commercial ELISA kit (#88-8350-77; Thermo Fisher Scientific) according to the manufacturer’s instructions. To obtain lung homogenates, lung tissues were homogenized in PBS with a tissue homogenizer (Precellys 24) and then centrifuged at 12,000 × *g* for 15 min at 4°C. To activate latent TGF-β1 expression in lung homogenates, 100 μL of homogenate was mixed with 20 μl of 1 N HCl and neutralized by adding 20 µL of 1.2 N NaOH/0.5 M HEPES.

### 2.12 Statistics

All experiments were repeated twice unless indicated otherwise. Data are presented as the mean ± SD of a representative experiment. Statistical significance was determined using one-way or two-way analysis of variance with Tukey’s multiple-comparison *post-hoc* tests. Statistical analyses were performed using GraphPad Prism 8.0 (GraphPad Prism Software, San Diego, CA, USA).

## 3 Results

### 3.1 Recovery of severe influenza pneumonia by LDRT

**Figure 1:**
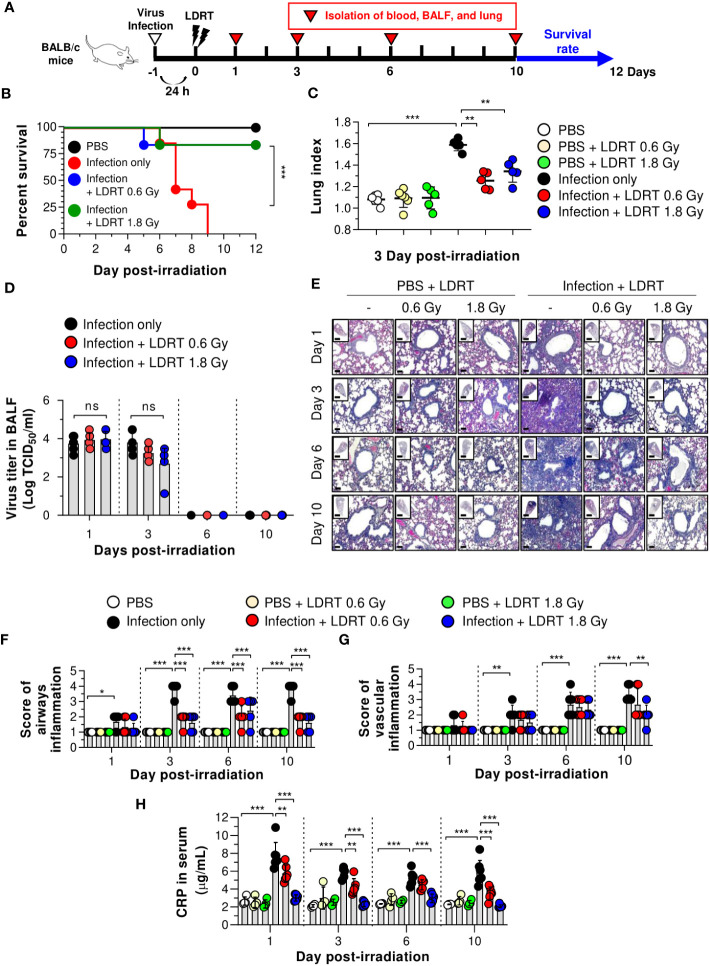
LDRT causes decreased lethality and pneumonia in a severe influenza infection mouse model. Mice were infected with 30 HAU of H1N1 (A/Solomon Islands/03/06) intranasally, followed by thoracic gamma-ray irradiation with 0.6 or 1.8 Gy at 1 day post-infection. Comparative effect of LDRT in an H1N1-infected mouse model. **(A)** Schematic representation of the experimental design. **(B)** Mouse survival was monitored daily for 12 days (6 mice/group). Mice were euthanized at body weight <80% of the initial weight. Statistical analysis was performed using two-way ANOVA in conjunction with Tukey’s test. ^***^ *p* < 0.001 **(C)** Mouse lung indexes were measured on day 3 after LDRT (5 mice/group). Statistical analysis was performed using one-way ANOVA in conjunction with Tukey’s test. ^**^ *p* < 0.01 and ^***^ *p* < 0.001. **(D)** Viral titers were determined in the BALF harvested at serial time points post-irradiation using a TCID_50_ assay (6 mice/group). Statistical analysis was performed using two-way ANOVA in conjunction with Tukey’s test. **(E)** Time course for representative H&E lung tissue staining post-infection followed by LDRT. Scale bars of whole slide images = 1000 μm (1× magnification) and scale bar of enlarged images = 100 μm (10× magnification). Histopathological airway **(F)** and vascular inflammation **(G)** scores for lung sections from mice. Lung sections were scored for airway and vascular inflammatory features from 0 to 5 by a board-certified pathologist in a blinded manner (5 mice/group). **(H)** Serum C-reactive protein (CRP) levels were analyzed using an automatic biochemical analyzer (PBS, n = 4; PBS + LDRT, n = 4; Infection, n =7; Infection + LDRT, n =7). **(D, F–H)** Data are presented as the mean ± SD. Statistical analysis was performed using two-way ANOVA in conjunction with Tukey’s test. ^*^ *p* < 0.05, ^**^ *p* < 0.01, and ^***^ *p* < 0.001. ns, not significant (*p* > 0.05).

We first investigated whether LDRT decreased pneumonia severity in an influenza-infected mouse model by conducting microbiological and immunological analyses. Mice were intranasally infected with 30 hemagglutination units (HAU) influenza A virus, followed by LDRT at a dose of 0.6 or 1.8 Gy on their thorax using a radioactive cesium-137 (^137^Cs) gamma irradiator 1 day post-infection because it has been proposed that LDRT will be most successful in the early stage of inflammation to prevent the development of ARDS caused by viral infection (11). A schematic of the experimental procedure is shown in **Figure 1A**. All control mice infected with the virus lost body weight rapidly and died eight days after infection. However, virus-infected mice groups treated with LDRT (infection + LDRT group) at 0.6 Gy or 1.8 Gy after infection exhibited only slight weight loss and 84% survival for 12 days. This finding established that LDRT could reduce pneumonia-related mortality caused by viral infection (**Supplementary Figure 1**; **Figure 1B**). The severity of pulmonary edema was evaluated using the lung index calculated as the ratio of lung weight to body weight in mice, as described previously (23). On day 3 after LDRT, the lung index of mice in the infection-only group was significantly higher than that in the control (phosphate-buffered saline, PBS) group. The group treated with LDRT had a markedly reduced lung index (**Figure 1C**). However, there were no differences in the number of viruses in the BALF between the infection-only and infection + LDRT groups (**Figure 1D**), suggesting that the recovery of viral pneumonia by LDRT was not due to the suppression of viral replication or direct inactivation of the virus.

To demonstrate whether LDRT inhibited lung inflammation, the pathological scores of airways or vascular inflammation on days 1, 3, 6, and 10 were investigated, as described previously (21). Consistent with the survival rate and lung index analyses, lungs from mice infected with the influenza A virus (infection-only group) showed marked pathological changes, including enhanced inflammatory cell infiltration, loss of alveolar architecture, and RBC leakage. However, a significant inflammation recovery was observed in the groups of infection + LDRT at 0.6 and 1.8 Gy compared to that in the infection-only group (**Figure 1E**). There was a significant increase in airway inflammation from day 1 to day 3 in the infection-only group. The airway score was significantly reduced from day 3 by LDRT at 0.6 and 1.8 Gy (**Figure 1F**). Conversely, vascular inflammation began to appear after 3 days and increased for up to 10 days in the infection-only group. A significant reduction in vascular inflammation appeared after 10 days of infection + LDRT at 1.8 Gy (**Figure 1G**). Finally, we measured the serum level of CRP, a marker of systemic inflammation in the serum (24). As expected, a high level of CRP in the blood was produced early after viral infection, and a considerable reduction in CRP levels by LDRT was observed on all days (**Figure 1H**). Although several hypotheses have stated that LDRT mediates the reduction of viral pulmonary pneumonia (25), we found that this was mainly due to the indirect reduction of lung inflammation by LDRT.

### 3.2 Modulation of influenza A virus-induced inflammatory cytokine levels by LDRT

**Figure 2:**
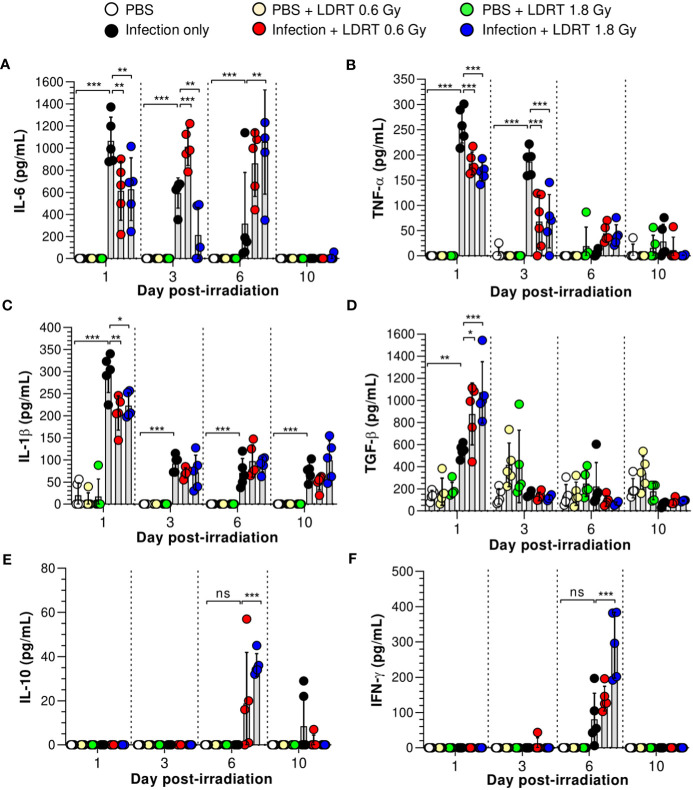
Time-course changes in cytokine levels in BALF after LDRT. Mice were infected with 30 HAU of H1N1 (A/Solomon Islands/03/06) intranasally (i.n.) followed by thoracic gamma-ray irradiation with 0.6 or 1.8 Gy at 1 day post-infection (d.p.i). BALF was obtained 1, 3, 6, and 10 days after thoracic irradiation. Levels of IL-6 **(A)**, TNF-α **(B)**, and IL-1β **(C)** in BALF were measured using the cytometric bead array (CBA) assay. **(D)** Levels of TGF-β in BALF were measured using ELISA. IL-10 **(E)** and IFN-γ **(F)** levels in BALF were measured using the CBA assays. These experiments were repeated twice (n = 5 mice/group in each experiment). All data are presented as the mean ± SD from the independent experiments. Statistical analysis was performed using two-way ANOVA in conjunction with Tukey’s test. ^*^ *p* < 0.05, ^**^ *p* < 0.01, and ^***^ *p* < 0.001. ns, not significant (p < 0.05).

We next investigated the alterations in pro- and anti-inflammatory cytokine production in the BALF of influenza A virus-infected mice. As shown in **Figure 2**, virus-infected mice exhibited elevated levels of primary cytokines (IL-6, TNF-α, IL-1β, and TGF-β) in the BALF on day 1, and IL-10 and IFN-γ were modestly raised after 6 days. However, after LDRT treatment, the pro-inflammatory cytokine (IL-6, TNF-α, and IL-1β) levels were significantly reduced on day 1, and this inhibitory effect against IL-6 and TNF-α was maintained until day 3 (**Figures 2A–C**). Conversely, the level of TGF-β, an anti-inflammatory cytokine, significantly increased on day 1 (**Figure 2D**). Furthermore, the levels of IL-6 and TNF-α, suppressed by LDRT, were reactivated on day 6 (**Figures 2A, B**), indicating that the effect of LDRT did not last until day 6, and the virus remaining in the lungs until day 3 was likely to reactivate immune responses. Similarly, the secondary cytokine (IFN-γ and IL-10) levels in the infection + LDRT group at 1.8 Gy were also higher than those in the infection-only group on day 6 (**Figures 2E, F**). IL-2 and IL-12p70 levels in BALF were not detected in any group (**Supplementary Figures 2A, B**). The levels of IL-6, and IL-1β in serum also suppressed by LDRT on day 1, but TNF-α and TGF-β unaffected (**Supplementary Figure 2C**). Both infection and LDRT had no effect on serum cytokine levels on day 3 (**Supplementary Figure 2D**).

Previous studies have speculated or reported that IL-10 is a critical element in modulating lung inflammation by LDRT (17). However, our data strongly suggest that IL-10 is involved in late anti-inflammatory activities after LDRT. By contrast, TGF-β, an initial response cytokine, is likely more important in the early anti-inflammatory mechanism of LDRT. Since pulmonary epithelial cells are one of the significant sources of TGF-β during viral infection (26), we investigated whether TGF-β is produced from the airway epithelium. Unexpectedly, a considerable level of TGF-β was found in the parenchyma region of the lung rather than the airway epithelium (**Supplementary Figure 3**).

### 3.3 Inhibition of immune cell recruitment by LDRT

**Figure 3:**
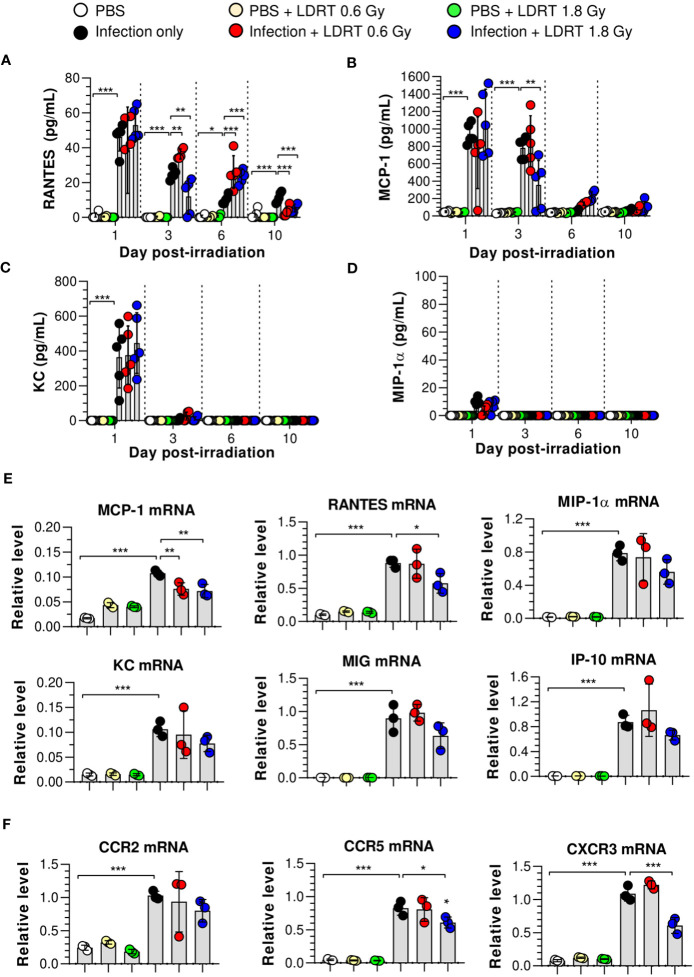
Time-course changes in chemokine levels in BALF and lung after LDRT. Mice were infected with 30 HAU of H1N1 (A/Solomon Islands/03/06) intranasally (i.n.) followed by gamma-ray thoracic irradiation with 0.6 or 1.8 Gy at 1 day post-infection (d.p.i). **(A–D)** Chemokine levels in BALF. BALF was obtained 1, 3, 6, and 10 days after thoracic irradiation. Levels of RANTES **(A)**, MCP-1 **(B)**, KC **(C)**, and MIP-1α **(D)** in BALF were measured using the CBA assays. These experiments were repeated twice (n = 5 mice/group in each experiment). Data are presented as the mean ± SD from the independent experiments. Statistical analysis was performed using two-way ANOVA in conjunction with Tukey’s test. ^*^ *p* < 0.05, ^**^ *p* < 0.01, and ^***^ *p* < 0.001. **(E, F)** Chemokine and chemokine receptor mRNA expression in whole lung tissue. Lung tissue was collected on day 3 following thoracic irradiation, and chemokine mRNA [*Ccl2* (MCP-1), *Ccl5* (RANTES), *Ccl3* (MIP-1α), *Cxcl1* (KC), *Cxcl9* (MIG), and *Cxcl10* (IP-10)] expression was quantified using qRT-PCR (n = 3 mice/group) **(E)**. Chemokine receptor mRNA (*Ccr2*, *Ccr5*, and *Cxcr3*) expression was quantified using qRT-PCR **(F)**. Gene expression levels are shown as fold change relative to uninfected mice (PBS) after normalization to 16S rRNA (housekeeping gene). Data are presented as the mean ± SD. Statistical analysis was performed using one-way ANOVA in conjunction with Tukey’s test. ^*^ *p* < 0.05, ^**^ *p* < 0.01, and ^***^ *p* < 0.001.

We hypothesized that the levels of chemokines regulated by LDRT were involved in alleviating lung inflammation induced by a viral infection and examined the expression of chemokines in the BALF and lungs of irradiated mice. The levels of regulated activation, normal T cell expressed and secreted (RANTES), and monocyte chemoattractant protein-1 (MCP-1) in BALF were significantly increased in the infection-only group on days 1 and 3. Keratinocytes-derived chemokine (KC) level was also increased on day 1 (**Figures 3A–C**). However, LDRT treatment at 1.8 Gy after infection suppressed the expression of RANTES and MCP-1 in BALF from day 3 (**Figures 3A, B**). Macrophage inflammatory protein-1α (MIP-1α) expression was not significantly increased following infection (**Figure 3D**). We further analyzed the mRNA expression of chemokines in lung tissues. Consistent with the previous results, the mRNA expression of all examined chemokines was not altered by LDRT on day 1 (data not shown), whereas *Ccl2* (MCP-1) and *Ccl5* (RANTES) expression levels were significantly reduced on day 3 (**Figure 3E**). The expression of chemokine receptors, such as *Ccr5* and *Cxcr3*, was also inhibited at a dose of 1.8 Gy (**Figure 3F**). Thus, chemokines expressed by influenza infection were downregulated during the LDRT-induced late anti-inflammatory phase.

**Figure 4:**
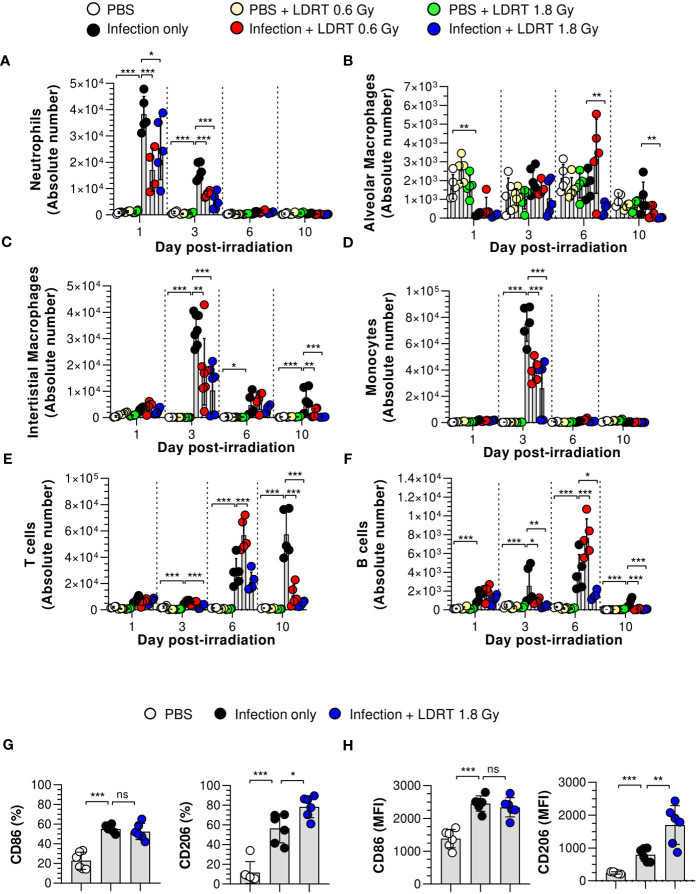
Prevention of immune cell infiltration and the polarization of M2 macrophages in the lungs after LDRT. Mice were infected with 30 HAU of H1N1 (A/Solomon Islands/03/06) intranasally (i.n.) followed by thoracic gamma-ray irradiation with 0.6 or 1.8 Gy at 1 day post-infection (d.p.i). Mouse BALF was collected 1, 3, 6, and 10 days after thoracic irradiation. BALF cell suspensions were stained with anti-CD45, anti-Ly6G, anti-CD11b, anti-CD11c, anti-MHC-II, anti-CD64, anti-CD24, and 7-aminoactinomycin D (7-AAD) antibodies. The stained cells were analyzed by absolute counts using flow cytometry. The cells were gated on single, live (7-AAD^-^), and CD45^+^ cells as neutrophils (**A**, Ly6G^+^), alveolar macrophages (**B**, Ly6G^-^MHC-II^+^CD64^+^CD24^-^CD11c^+^ CD11b^low^), interstitial macrophages (**C**, Ly6G^-^MHC-II^+^CD64^+^CD24^-^CD11c^low^CD11b^+^), monocytes (**D**, Ly6G^-^ Ly6G^-^CD11c^+^MHC-II^-^CD64^+^CD11b^+^), T cells (**E**, Ly6G^-^CD11c^-^CD11b^-^MHC-II^-^CD24^-^), and B cells (**F**, Ly6G^-^CD11c^-^CD11b^-^MHC-II^+^CD24^+^). **(G, H)** BALF was harvested on day 3 after thoracic irradiation, and M1 and M2 surface markers of the macrophage **(G)** and monocytes **(H)** in BALF were analyzed using flow cytometry following staining with anti-CD45, anti-CD11b, anti-CD11c, anti-MHC-II, anti-CD64, anti-CD86, anti-CD206, and live/dead cell staining. CD45^+^CD11b^+^CD11c^+^MHC-II^+^CD24^-^CD64^+^ cells were defined as macrophages in BALF. Percentage of CD86^+^ or CD206^+^ or mean fluorescence intensity (MFI) in BALF macrophages and monocytes. These experiments were repeated twice (n = 5 - 6 mice/group in each experiment). All data are presented as the mean ± SD from the independent experiments. Statistical analysis was performed using two-way ANOVA in conjunction with Tukey’s test. ^*^ *p* < 0.05, ^**^ *p* < 0.01, and ^***^ *p* < 0.001. ns, not significant (p < 0.05).

Next, we hypothesized that LDRT-regulated chemokines might inhibit late-stage immune cell migration to the lung. We analyzed the effect of LDRT on the immune cell composition of BALF from mice infected with the influenza virus. The gating strategy is shown in **Supplementary Figure 4**. The population of alveolar macrophages (Ly6G^-^MHC-II^+^CD64^+^CD24^-^CD11c^+^CD11b^low^) was reduced following viral infection on day 1, which then recovered and was maintained from day 3 (**Figure 4B**). The decrease in the population of alveolar macrophages due to infection did not change after LDRT until day 3. On days 6 and 10, we found a slightly reduced number of alveolar macrophages in the infection + LDRT 1.8 Gy group only (**Figure 4B**). Interstitial macrophages (Ly6G^-^MHC-II^+^CD64^+^CD24^-^CD11c^low^CD11b^+^) and monocytes (Ly6G^-^CD11c^+^MHC-II^-^CD64^+^CD11b^+^) began to accumulate on day 3 while significantly reduced by LDRT at 0.6 and 1.8 Gy on day 3 (**Figures 4C, D**). T cells (Ly6G^-^CD11c^-^CD11b^-^MHC-II^-^CD24^-^) and B cells (Ly6G^-^CD11c^-^CD11b^-^MHC-II^+^CD24^+^) were subsequently recruited into the BALF by a viral infection on days 3 to 10, but the increased T cells and B cells following by infection strongly inhibited by LDRT in the 1.8 Gy group on days 6 and 10, respectively (**Figures 4E, F**). A surprising finding was that the neutrophil population (Ly6G^+^) rapidly increased on day 1 after viral infection, which was significantly reduced by LDRT at 0.6 and 1.8 Gy on days 1 and 3 (**Figure 4A**). LDRT effectively inhibited the infiltration of immune cells into the respiratory tract following viral infection at the late inflammation stage. However, alveolar macrophages, which are tissue-resident cells, were not directly affected. In contrast, blockage of neutrophil infiltration was not due to chemokine downregulation, but other indirect or direct LDRT effects are involved.

We next examined the number of leukocytes in the blood using an automatic hematology analyzer. The counts of white blood cells, neutrophils, and monocytes were increased following infection on days 1, 3, and 10 (**Supplementary Figure 5**). These cell counts are considered parameters of severe neutrophilia and monocytosis; however, they were improved markedly by LDRT. These results indicate that LDRT suppresses neutrophilia and monocytosis induced by the lethal attack of the influenza A virus. In addition, it suppresses immune cell infiltration into the respiratory tract.

Although the number of alveolar macrophages was not significantly changed after LDRT, previous reports suggested that macrophage polarization plays an essential role in the pathogenesis of pulmonary pneumonia caused by viral infection (27). In addition, several reports have suggested that radiation polarizes macrophages toward an M2-like phenotype, known as alternatively activated or anti-inflammatory macrophages (28). Thus, we examined the M1/M2 phenotype changes induced by LDRT. CD45^+^CD11b^+^CD11c^+^MHC-II^+^CD64^+^ cells were used to define macrophages in the BALF (**Supplementary Figure 6**). The upregulated expression of CD86 (M1 marker) in both macrophages (**Figure 4G**) and monocytes (**Figure 4H**) of the BALF was maintained after LDRT. In contrast, the expression of CD206 (M2 marker) in both cell types was significantly increased in the infection + LDRT 1.8 Gy group compared to that in the infection-only group. These results suggest that the enhancement of the M2 phenotype is due to a preference for recruiting M2 macrophages to the lungs over M1 macrophages/monocytes or an enhancement of M2 polarization over M1 polarization after LDRT.

### 3.4 M2 polarization by LDRT-induced TGF-β production

**Figure 5:**
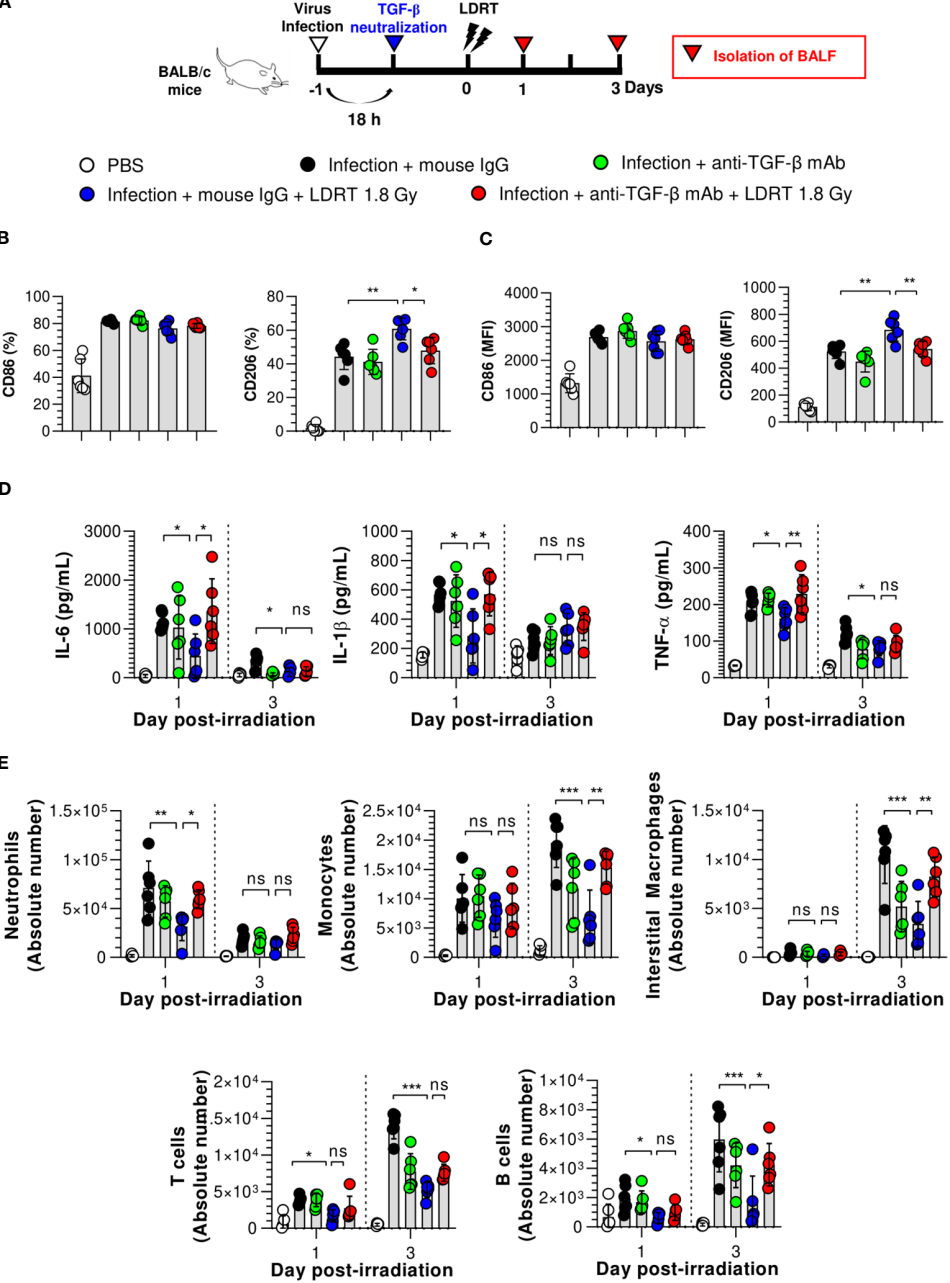
TGF-β reduces the anti-inflammatory effects of LDRT. Mice were intraperitoneally administered anti-TGF-β monoclonal antibody (200 μg/mouse) or mouse IgG antibody (isotype control) 4 h before thoracic irradiation (1.8 Gy), and mouse BALF was obtained on days 1 and 3. **(A)** Schematic representation of the experimental design. **(B, C)** Inhibition of M2 macrophage **(B)** and monocytes **(C)** polarization by TGF-β neutralization. **(D)** Inhibition of pro-inflammatory cytokines (IL-6, IL-1β, and TNF-α) in the BALF by TGF-β neutralization. Levels of pro-inflammatory cytokines were analyzed using the CBA assay. **(E)** Enhancement of immune cell infiltration by TGF-β neutralization. The absolute number of each immune cell in the single-cell suspension of BALF was analyzed using flow cytometry. Data are presented as the mean ± SD (n = 6 mice/group). Statistical analysis was performed using two-way ANOVA in conjunction with Tukey’s test. ^*^ *p* < 0.05, ^**^ *p* < 0.01, and ^***^ *p* < 0.001. ns, not significant (*p* > 0.05).

Since TGF-β, the early-response cytokine observed after LDRT (**Figure 2D**), is a well-known key regulator of M2 polarization, we investigated whether a blockade of TGF-β expression led to the suppression of the M2 phenotype. Twenty hours after the influenza virus infection, mice were intraperitoneally injected with anti-TGF-β mAb to neutralize TGF-β, followed by LDRT treatment (**Figure 5A**). The expression of CD86 was not significantly changed in BALF macrophages (**Figure 5B**) and monocytes (**Figure 5C**), even after treatment with the anti-TGF-β mAb. However, the increased expression of CD206 by LDRT after infection decreased in the anti-TGF-β mAb-treated group to a level similar to that of the infection + mouse IgG mAb (isotype control) group. These results suggest that the increase in the M2 phenotype in virus-infected mice might be due to LDRT-induced TGF-β-mediated M2 polarization or migration.

### 3.5 TGF-β is a key modulator of LDRT

Next, we demonstrated that TGF-β is a key modulator of LDRT by analyzing whether the blockade of TGF-β directly affects LDRT-induced anti-inflammatory action in the lungs. Mice were intraperitoneally injected with anti-TGF-β mAb 20 h after viral infection, as described above. In addition, the production of cytokines and chemokines and the population of immune cells were analyzed at 1 and 3 days after LDRT. A schematic of the experimental procedure is shown in **Figure 5A**. As shown in **Figure 5D**, the reduced levels of early-response cytokines (IL-6, IL-1β, and TNF-α) following LDRT after infection on both day 1 and 3 were increased in the anti-TGF-β mAb-treated group, which was similar to the levels in virus-infected mice without LDRT (infection + mouse IgG group). However, the reduced chemokine responses (KC, RANTES, and MCP-1) by LDRT tended to increase in the anti-TGF-β mAb-treated group. They did not change significantly on days 1 and 3 (**Supplementary Figure 7**).

Because the cytokines and chemokines downregulated by LDRT inhibited immune cell migration into the lungs, we assessed whether TGF-β neutralization increased immune cell migration. Early-response immune (neutrophils) cell counts that were reduced on day 1 and late-response immune cell (monocytes, interstitial macrophages, and B cells) counts that were reduced on day 3 by LDRT increased in the anti-TGF-β mAb-treated group to a level similar to that in the untreated mice (infection + mouse IgG group, **Figure 5E**). These data strongly suggest that early induction of TGF-β production by LDRT suppresses viral pneumonia progression by downregulating inflammation, blocking immune cell migration, and promoting M2 polarization at the site of infection.

### 3.6 Preclinical safety of LDRT

**Figure 6:**
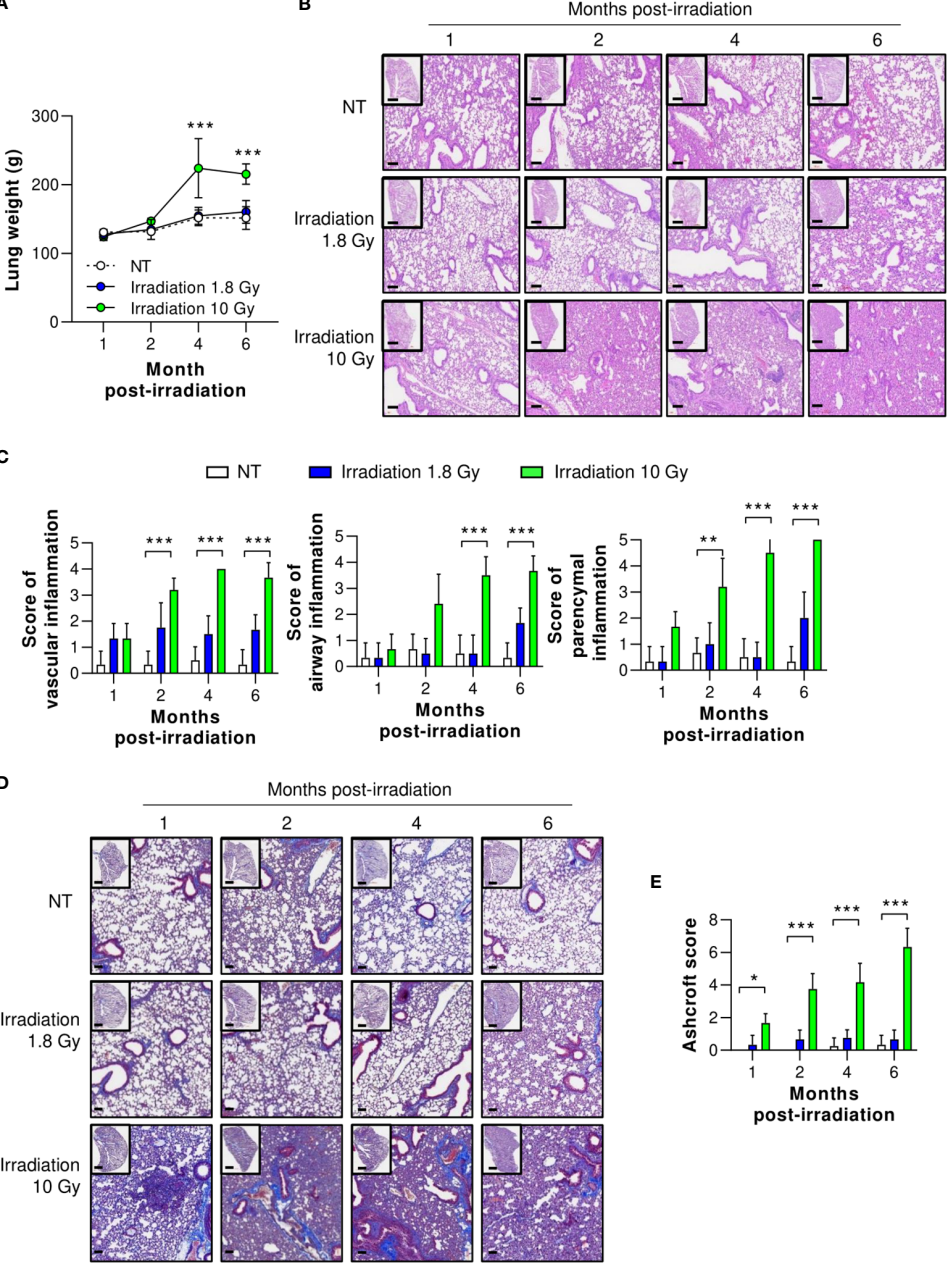
Preclinical safety of LDRT. Mice were irradiated on the thorax at doses of 1.8 or 10 Gy. Lung tissues were harvested 1, 2, 4, and 6 months post thoracic irradiation. **(A)** Lung weight change was recorded for six months after irradiation. **(B)** Representative H&E lung tissue staining post thoracic irradiation. Scale bars of whole slide images = 1000 μm (1× magnification) and scale bar of enlarged images = 100 μm (10× magnification). **(C)** H&E-stained lung sections were scored for airway, vascular, and parenchymal inflammatory features from 0 to 5 by a broad-certified, blinded pathologist (5 mice/group). **(D)** Masson’s trichrome-stained lung sections for lung fibrosis. Scale bars of whole slide images = 1000 μm (1× magnification) and scale bar of enlarged images = 100 μm (10× magnification). **(E)** Ashcroft scores were measured to quantify collagen contents (5 mice/group). The scores ranged from 0 (normal lung tissue) to 8 (total fibrous obliteration of the tissue). Data are presented as the mean ± SD (n = 5 mice/group). Statistical analysis was performed using two-way ANOVA in conjunction with Tukey’s test. ^*^ *p* < 0.05, ^**^ *p* < 0.01, and ^***^ *p* < 0.001.

Although many reviews agree on the efficacy of LDRT for treating viral pneumonia, the safety of this treatment is of great concern (29–32). Therefore, we investigated protection against long-term radiation-induced pneumonitis (RIP) and lung fibrosis, which are immunologically related to high levels of TGF-β expression 1, 2, 4, and 6 months after thoracic irradiation. At six months, mice treated with irradiation at 1.8 Gy showed no increase in lung tissue weight and no histological changes, including vascular inflammation, airway inflammation, and parenchymal inflammation (**Figures 6A–C**). However, mice treated with irradiation at 10 Gy, the positive control group, showed significantly higher inflammation than the normal group two months after irradiation. They also exhibited increased lung weight due to hypertrophy (**Figure 6A**). When the pathological scores of the airway, lung vascular, and parenchymal inflammation were calculated, they were slightly, but not significantly, increased by irradiation at 1.8 Gy. However, we observed significant increases in all inflammation scores in the group treated with irradiation at 10 Gy (**Figures 6B, C**).

To evaluate long-term pulmonary fibrosis, Masson’s trichrome (MT) staining of the lungs was performed and levels of fibrosis-induced cytokines were measured 1, 2, 4, and 6 months after irradiation of the thorax. As shown in **Figures 6D, E**, collagen deposition staining (blue color) was performed, and lung fibrosis (Ashcroft score) was calculated. In the group irradiated with 10 Gy, significant lung fibrosis was observed one month after irradiation, and its severity continued to increase. By contrast, no significant difference in lung fibrosis was observed between the group irradiated with 1.8 Gy and the non-treated group. Although these data were evaluated in mice, they imply that LDRT had no significant radiation-induced long-term adverse effects.

## 4 Discussion

This study determined one of the probable mechanisms of LDRT in pneumonia relief via immunological alterations in influenza A-infected mice. We observed that LDRT effectively and directly reduced influenza-induced pulmonary inflammation by enhancing TGF-β levels in the lungs more than other anti-cytokines in the early phase of infection. Our novel key finding is that TGF-β plays a vital role in LDRT-mediated anti-inflammatory action against influenza-induced pneumonia and can considerably reduce or delay CS. Therefore, LDRT or recombinant TGF-β could be an effective treatment method for preventing pulmonary viral infections, including COVID-19.

A recent study by Meziani et al. reported that LDRT protects against influenza A infection by inducing an increase in the number of IL-10-producing nerve- and airway-associated macrophages (17). However, we observed that TGF-β was a key cytokine in the initial phase of LDRT-mediated anti-inflammatory action, whereas the IL-10 level seemed to increase indirectly in the late phase. TGF-β, a multitasking cytokine with complex roles in the immune system, is considered a master regulator of radiotherapy to maximize anticancer immunity (33). The most crucial role of TGF-β in the immune system is maintaining immune homeostasis in both self and benign antigens (34). One anti-inflammatory role of TGF-β is to inhibit the infiltration of immune cells into sites of infection or inflammation (35). This inhibitory effect on immune cell infiltration is linked to the downregulation of chemoattractant production and induction of their clearance through apoptosis in the early stages of acute lung injury (36, 37). However, according to previous studies, LDRT does not seem to inhibit neutrophil infiltration because the timing of TGF-β expression and the suppression timing of chemoattractant do not match. Nevertheless, the blockage of TGF-β showed the enhancement of immune cell infiltration; we concluded that TGF-β directly or indirectly modulates immune cell infiltration and inflammation. Other studies reported that the effect of radiation on immune cell infiltration is linked to vascular homeostasis through the downregulation of adhesion molecules (38, 39). However, these previous findings may not explain the efficacy of LDRT through TGF-β, as the suppression of anti-immune responses and immune cell infiltration by LDRT is too drastic and rapid. Therefore, further LDRT immunological mechanistic studies must clarify and support LDRT clinical trials.

Altering the macrophage phenotype effectively modulates the host immune response during radiotherapy (40, 41). Among the various anti-inflammatory mechanisms of LDRT for COVID-19, M2 macrophage induction is a promising mechanism for treating severe pulmonary injury by suppressing CS (42). M2 macrophages participate in tissue healing and remodeling by clearing apoptotic cells, contributing to the resolution of inflammation (43). Furthermore, M2 macrophages protect the host from long-term progression to fibrotic lung disease during SARS-CoV-2 infection (44, 45). Various cytokines and the microenvironment can switch the macrophage polarization state. TGF-β is a well-known cytokine that induces M2-like macrophage polarization by activating the canonical TGF-β/Smad-mediated signaling (46, 47). Although the specific mechanism by which LDRT causes phenotypic transformation into M2 macrophages remains unclear, we postulated that higher TGF-β levels following LDRT caused the M2 polarization of macrophages. Our results showed that M2 macrophage count increased following LDRT after influenza infection, but these phenotypic changes were abrogated in the TGF-β-neutralized group using an anti-TGF-β antibody. Therefore, LDRT drives the M2-like phenotype via TGF-β induction, which may resolve pneumonia pathology by maintaining tissue homeostasis. The detailed mechanisms underlying immunological alterations in the late phase of infection following LDRT, particularly the correlation between secondary cytokines and T cell-mediated immunity, should be elucidated in future studies.

**Figure 7:**
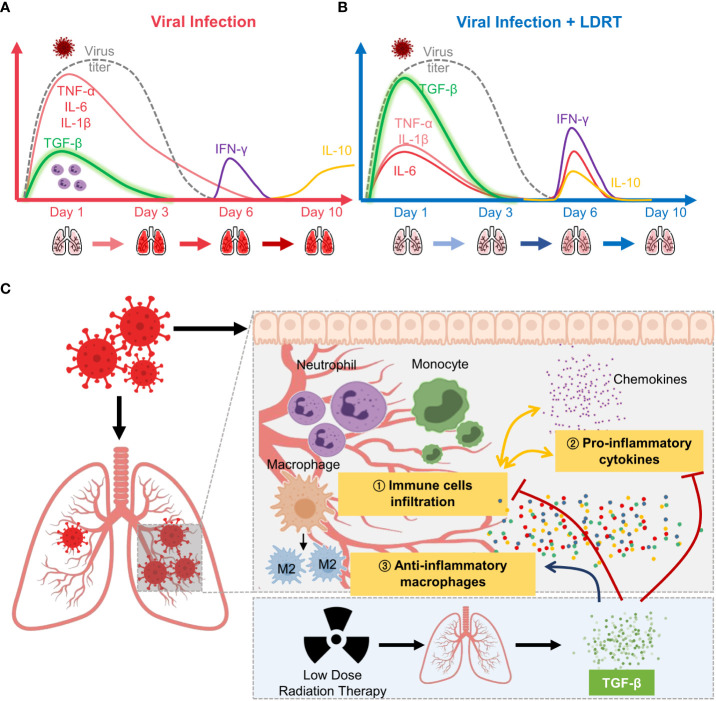
Overview of low-dose radiation therapy (LDRT) for virus-associated pneumonia. **(A)** Lethal-dose viral infection caused excessive inflammatory responses, such as increased production of primary cytokines (TNF-α, IL-6, and IL-1β; red line) and reduced TGF-β (green line) levels in the early phase of infection. Viral replication did not persist until day 6 (broken grey line), but levels of secondary cytokines (IFN-γ, purple line; IL-10, yellow line) increased on days 6 to 10. **(B)** By contrast, LDRT treatment after viral infection decreased the level of primary cytokines with high levels of TGF-β in the early phase, and the increase in the levels of cytokines (IL-6, IL-10, and IFN-γ) was eliminated by day 10. **(C)** Following an increase in TGF-β production, LDRT orchestrated anti-inflammatory and immune responses, including the reduced immune cell infiltration and chemokine responses and phenotypic changes of macrophages into M2. These anti-inflammatory effects of LDRT can alleviate the severity of the disease caused by viral infection.

In summary, LDRT on the thorax increased TGF-β production in the early phase of influenza infection, thereby inducing broad-spectrum anti-inflammatory actions, inhibiting immune cell infiltration and primary cytokine production, in addition to increasing M2 macrophage counts. Furthermore, these immunological alterations reduced the clinical signs of pneumonia (**Figure 7**). Our findings contribute to a better understanding of the mechanism of LDRT as a treatment for CS and ARDS in viral pneumonia.

## Data availability statement

The original contributions presented in the study are included in the article/**Supplementary Material**, further inquiries can be directed to the corresponding author/s.

## Ethics statement

The animal study was reviewed and approved by Korea Atomic Energy Institute-Institutional Animal Care and Use Committee (KAERI-IACUC-2021-003).

## Author contributions

KA and HS conceived and designed the overall study. H-YS, FC, H-RP, JH, HJ, YK, and KA conducted experiments and acquired data. H-YS, FC, H-RP, E-BB, YK, and MK performed statistical analysis. H-YS, FC, KA, and HS contributed to manuscript writing and editing. All authors contributed to the article and approved the submitted version.
