# Supplementary material for: Low-dose radiation therapy suppresses viral pneumonia by enhancing broad-spectrum anti-inflammatory responses via transforming growth factor-β production
Source: Front Immunol. 2023 May 25;14:1182927. doi: 10.3389/fimmu.2023.1182927 (PMC10248130; doi:10.3389/fimmu.2023.1182927)
Supplement: Supplementary file 1 [file DataSheet_1.docx]

**Low-dose radiation therapy suppresses viral pneumonia by enhancing broad-spectrum anti-inflammatory responses via transforming growth factor-β production**

Ha-Yeon Song^1†^, Fengjia Chen^1†^, Hae-Ran Park^1^, Jeong Moo Han^1,2^, Hyun Jung Ji^1,3^, Eui-Baek Byun^1^, Yeongkag Kwon^1^, Minkyu Kim^1^, Ki Bum Ahn^1,4*^, Ho Seong Seo^1,5*^

**Author affiliations**

*^1^Research Division for Radiation Science, Advanced Radiation Technology Institute, Korea Atomic Energy Research Institute, Jeongeup, Republic of Korea, ^2^Department of Biotechnology, College of Life Science and Biotechnology, Korea University, Seoul, Republic of Korea, ^3^Department of Oral Microbiology and Immunology, DRI, and BK21 Plus Program, School of Dentistry, Seoul National University, Seoul, Republic of Korea, ^4^Animal Production and Health Laboratory, Joint FAO/IAEA Centre for Nuclear Applications in Food and Agriculture, Department of Nuclear Sciences and Applications, International Atomic Energy Agency, Seibersdorf, Austria, ^5^Department of Radiation Science, University of Science and Technology, Daejeon, Republic of Korea*

**^*^Correspondence:**

Ki Bum Ahn

[ahnkb@kaeri.re.kr](mailto:ahnkb@kaeri.re.kr)

Ho Seong Seo

[hoseongseo@kaeri.re.kr](mailto:hoseongseo@kaeri.re.kr)

^†^**Co-first authors:**

These authors equally contributed to this work.

**Supplemental Table 1. Primers used in this study**

| **Gene name** | **Sequences** |
| --- | --- |
| *Ccl2* | 5’- CCC AAT GAG TAG GCT GGA GA -3’ |
|  | 5’- AGA CCT TAG GGC AGA TGC AG -3’ |
| *Ccl5* | 5’- CCC TCA CCA TCA TCC TCA CT-3’ |
|  | 5’- CCT TCG AGT GAC AAA CAC GA-3’ |
| *Ccl3* | 5’- ACC ATG ACA CTC TGC AAC CA-3’ |
|  | 5’- CCC AGG TCT CTT TGG AGT CA-3’ |
| *Cxcl1* | 5’- GCT GGG ATT CAC CTC AAG AA-3’ |
|  | 5’- TGG GGA CAC CTT TTA GCA TC-3’ |
| *Cxcl9* | 5’- CGC TGT TCT TTT CCT CTT GG-3’ |
|  | 5’- TTT TTC CCC CTC TTT TGC TT-3’ |
| *Cxcl10* | 5’- AAG TGC TGC CGT CAT TTT CT-3’ |
|  | 5’- TTC ATC GTG GCA ATG ATC TC-3’ |
| *Ccr2* | 5’- TGT TAC CTC AGT TCA TCC ACG GCA-3’ |
|  | 5’- AGC CCT GTG CCT CTT CTT CTC ATT-3’ |
| *Ccr5* | 5’- CGT TTG ACC ATG TGT TTT CG -3’ |
|  | 5’- ACA CCC TGT TTC GCT GTA GG -3’ |
| *Cxcr3* | 5’- CTG CCC ACA ATG GAA GAG TT -3’ |
|  | 5’- TGC TAG ATG CCT CGG ACT TT -3’ |
| *Actb* | 5’- GAC CTG TAC GCC AAC ACA GT-3’ |
|  | 5’- CCA GGG CAG TGA TCT CCT TC-3’ |

**Supplemental Figures**


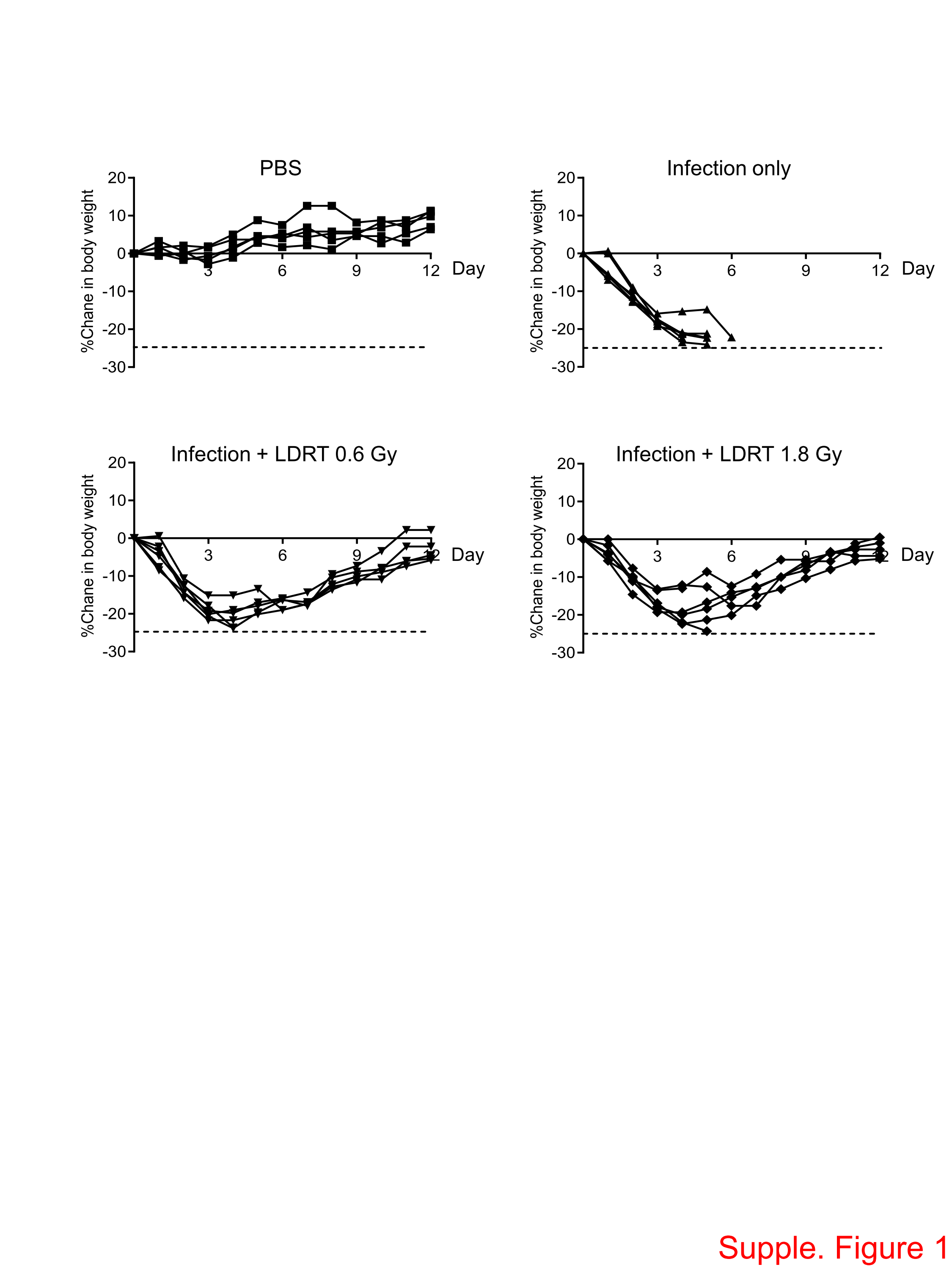


**Supplemental Figure 1. LDRT prevents weight loss in a severe influenza infection mouse model.** Mice were infected with 30 HAU of H1N1 (A/Solomon Islands/03/06) intranasally (i.n.) followed by thoracic gamma ray irradiation with 0.6 or 1.8 Gy 1 day post infection (d.p.i). Weight changes in mice were monitored daily for 12 days. Mice were euthanized at body weight <80% of initial weight (PBS, n = 5; Infection only, n = 6; Infection + LDRT 0.6 Gy, n = 6; Infection + LDRT 1.8 Gy, n = 6).


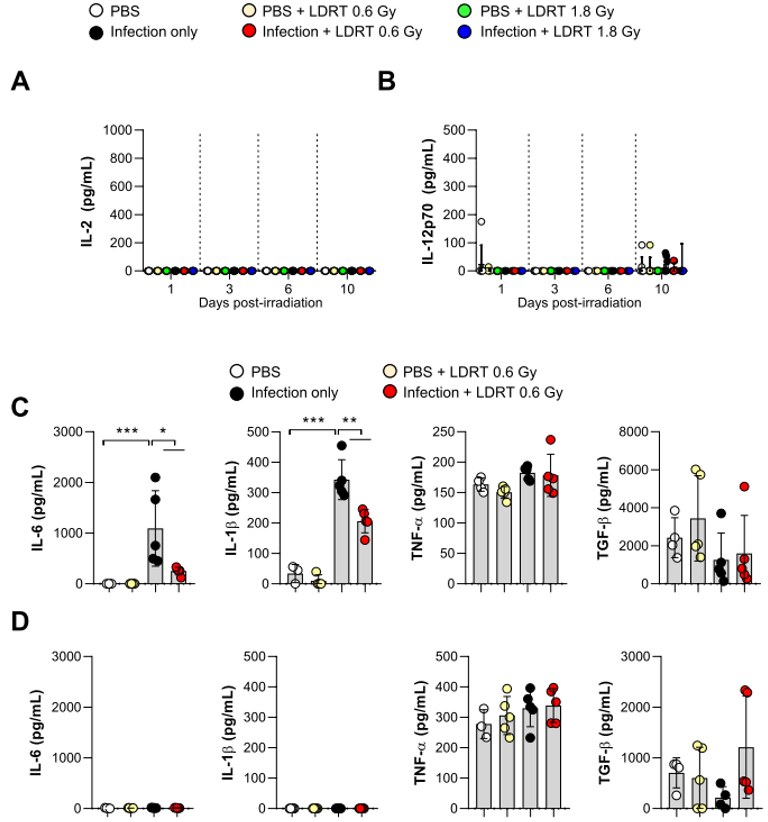


**Supplemental Figure 2. Time-course** **changes in various cytokines levels in BALF and serum after LDRT.** Mice were infected with 30 HAU of H1N1 (A/Solomon Islands/03/06) intranasally (i.n.) followed by thoracic gamma ray irradiation with 0.6 or 1.8 Gy 1 day post infection (d.p.i). BALF was harvested on days 1, 3, 6, and 10 post thoracic irradiation. The levels of IL-2 (A) and IL-12p70 (B) in BALF were measured using the cytometric bead array (CBA) assays. The levels of IL-6, IL-1β, TNF-α, and TGF-β in serum were determined using commercial ELISA kits 1 day (C) and 3 days (D) after thoracic irradiation. Data are presented as the mean ± SD (n = 5 mice/group).

**
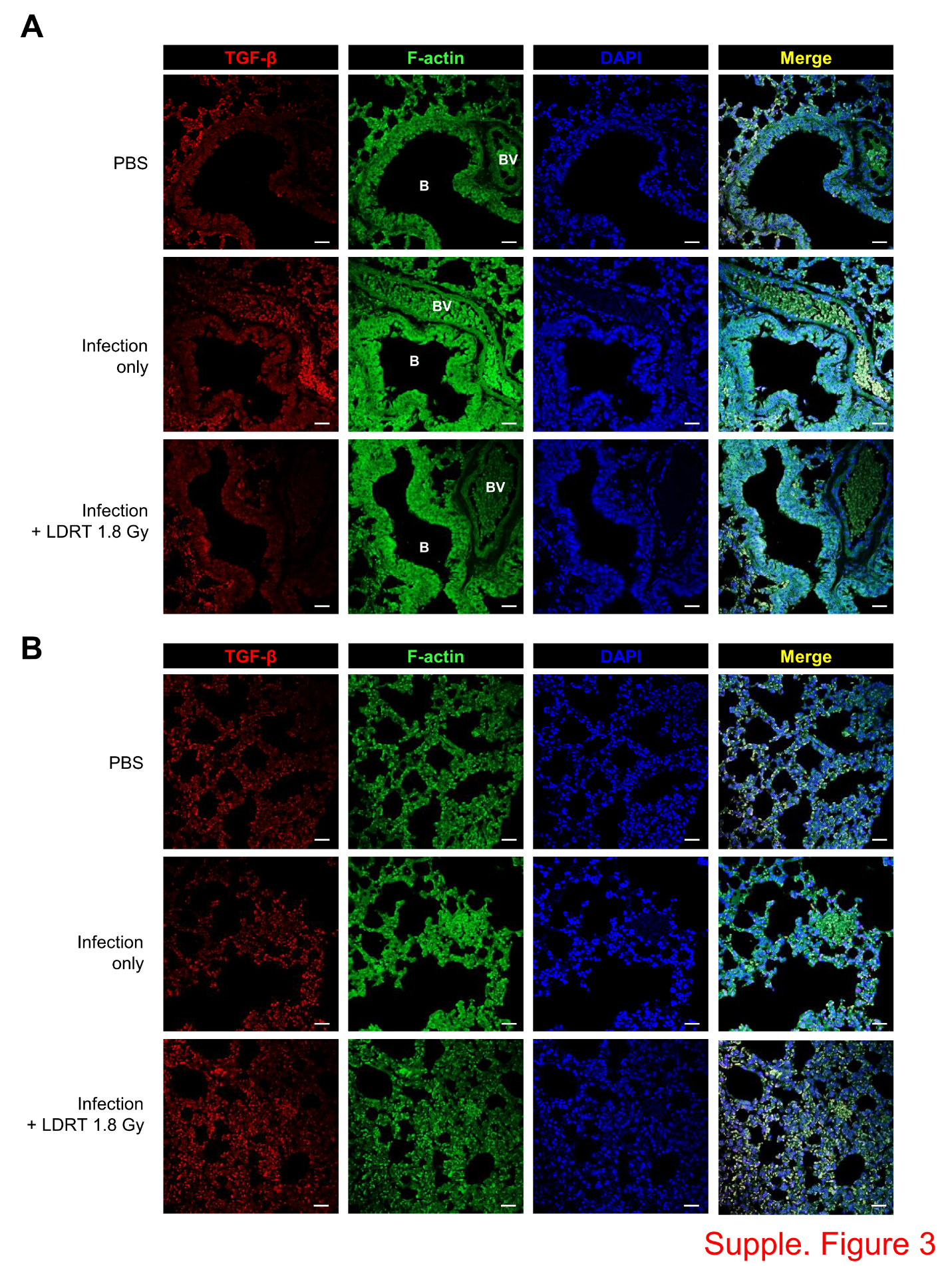
**

**Supplemental Figure 3. Localization of TGF-β in lung after LDRT.** Mice were infected with 30 HAU of H1N1 (A/Solomon Islands/03/06) intranasally (i.n.) followed by thoracic gamma ray irradiation with 0.6 or 1.8 Gy 1 day post infection (d.p.i). Lung tissues were harvested on day 1 post thoracic irradiation. Lung tissues were assessed by immunofluorescence staining for TGF-β. Representative staining images of the airway (A) or parenchyma (B) region. Representative fluorescein showed TGF-β (red), F-actin (green) with nuclear DAPI (blue), and was obtained by confocal laser scanning microscopy. Scale bar = 50 μm. B, bronchiole; BV, blood vessel.


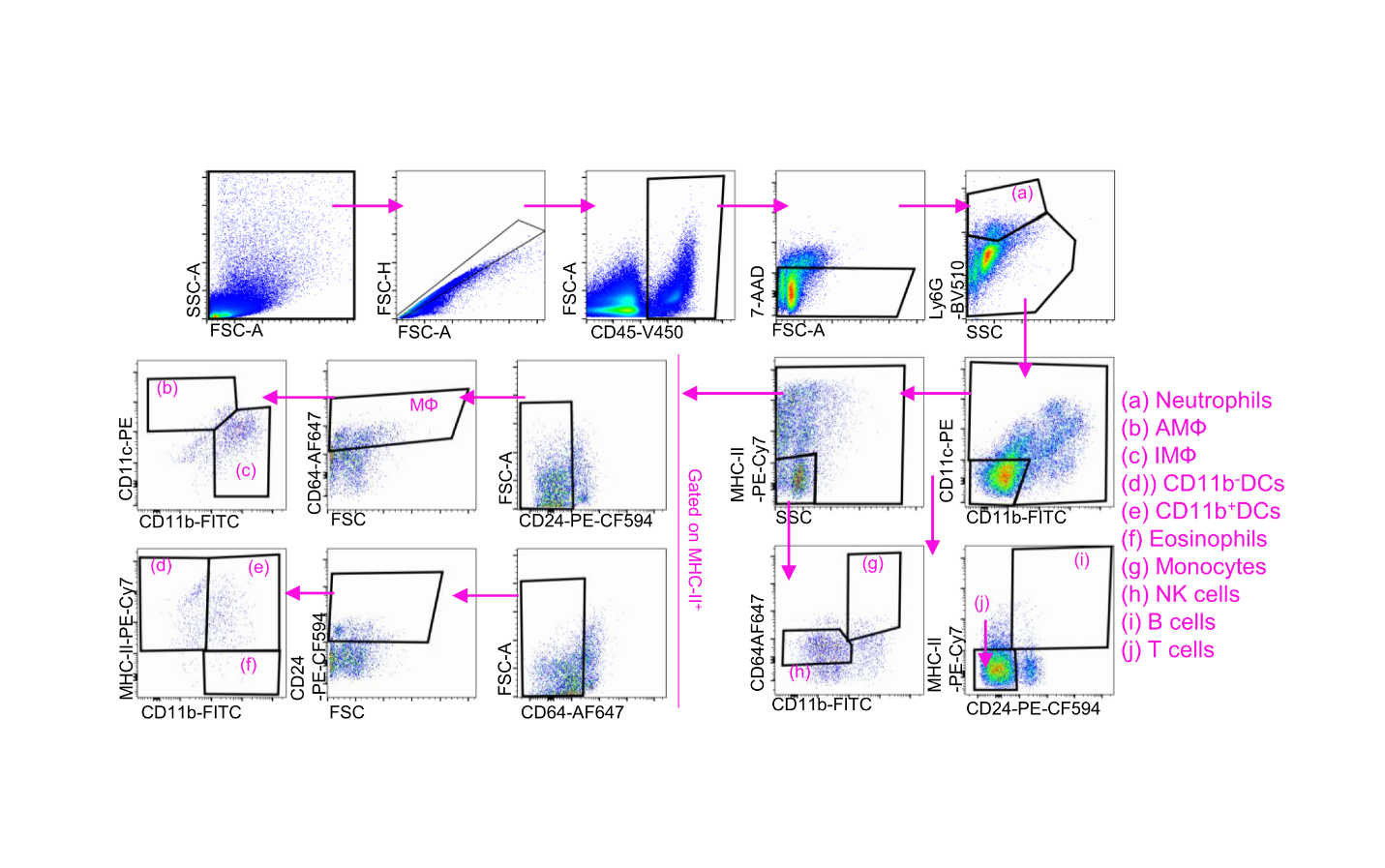


**Supplemental Figure 4. Gating strategy of immune cells in BALF.** BALF cell suspensions were stained with anti-CD45, anti-Ly6G, anti-CD11b, anti-CD11c, anti-MHC-II, anti-CD64, anti-CD24, and 7-aminoactinomycin D (7-AAD). The stained cells were analyzed using flow cytometry. Cells were gated on single, live (7-AAD^-^), and CD45^+^ cells. Ly6G^+^ as neutrophils (a), Ly6G^-^MHC-II^+^CD64^+^CD24^-^CD11c^+^CD11b^low^ as alveolar macrophages (AMФ, b), Ly6G^-^MHC-II^+^CD64^+^CD24^-^CD11c^low^CD11b^+^ cells as interstitial macrophages (IMФ, c), Ly6G^-^MHC-II^+^CD64^-^CD24^+^CD11b^+^ cells as CD11b^+^ dendritic cells (DCs, d), Ly6G^-^MHC-II^+^CD64^-^CD24^+^CD11b^-^ as CD11b-DCs (e), Ly6G^-^CD64^-^CD24^+^MHC-II^-^CD11b^+^ cells as eosinophils (f), Ly6G^-^CD11c^+^MHC-II^-^CD64^+^CD11b^+^ cells as monocytes (g), Ly6G^-^CD11c^+^MHC-II^-^CD64^low^CD11b^low^ cells as natural killer (NK) cells (h), Ly6G^-^CD11c^-^CD11b^-^MHC-II^+^CD24^+^ cells as B cells (i), and Ly6G^-^CD11c^-^CD11b^-^MHC-II^-^CD24^-^ cells as T cells (j).


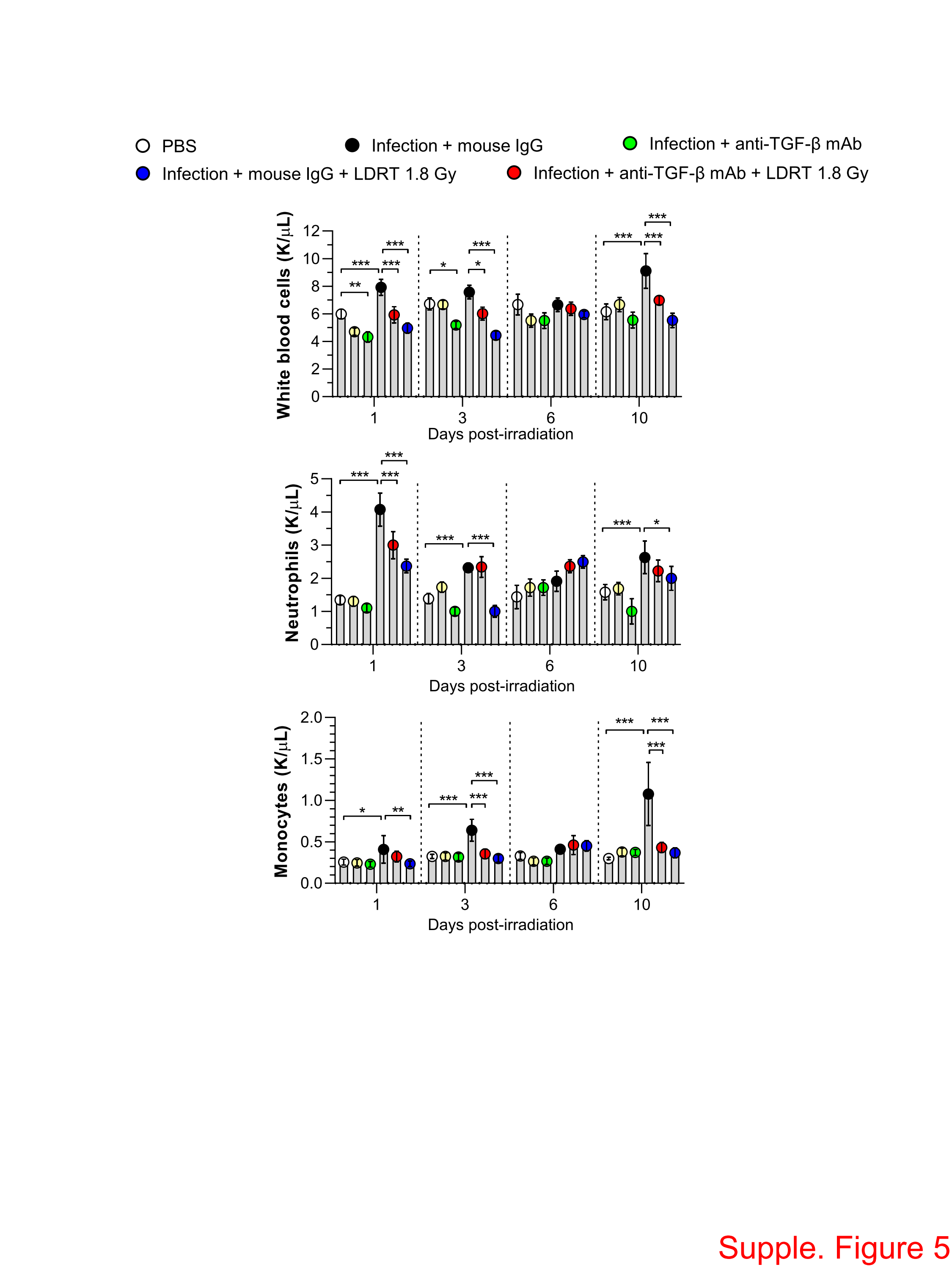


**Supplemental Figure 5. Changes in mouse peripheral blood cell counts after LDRT.** Mice were infected with 30 HAU of H1N1 (A/Solomon Islands/03/06) intranasally (i.n.) followed by thoracic gamma ray irradiation with 0.6 or 1.8 Gy 1 day post-infection (d.p.i). Blood samples were collected using K_3_EDTA tubes from the eyes under anesthesia on days 1, 3, 6, and 10 after thoracic irradiation. Total white blood cell (WBCs), neutrophil, and monocyte counts in the blood were determined using a Hemavet animal blood cell counter. Data are presented as the mean ± SD (n = 4 mice per group). Statistical analysis was performed using two-way ANOVA in conjunction with Tukey’s test. ^*^*p* < 0.05, ^**^*p* < 0.01, and ^***^*p* < 0.001.

**
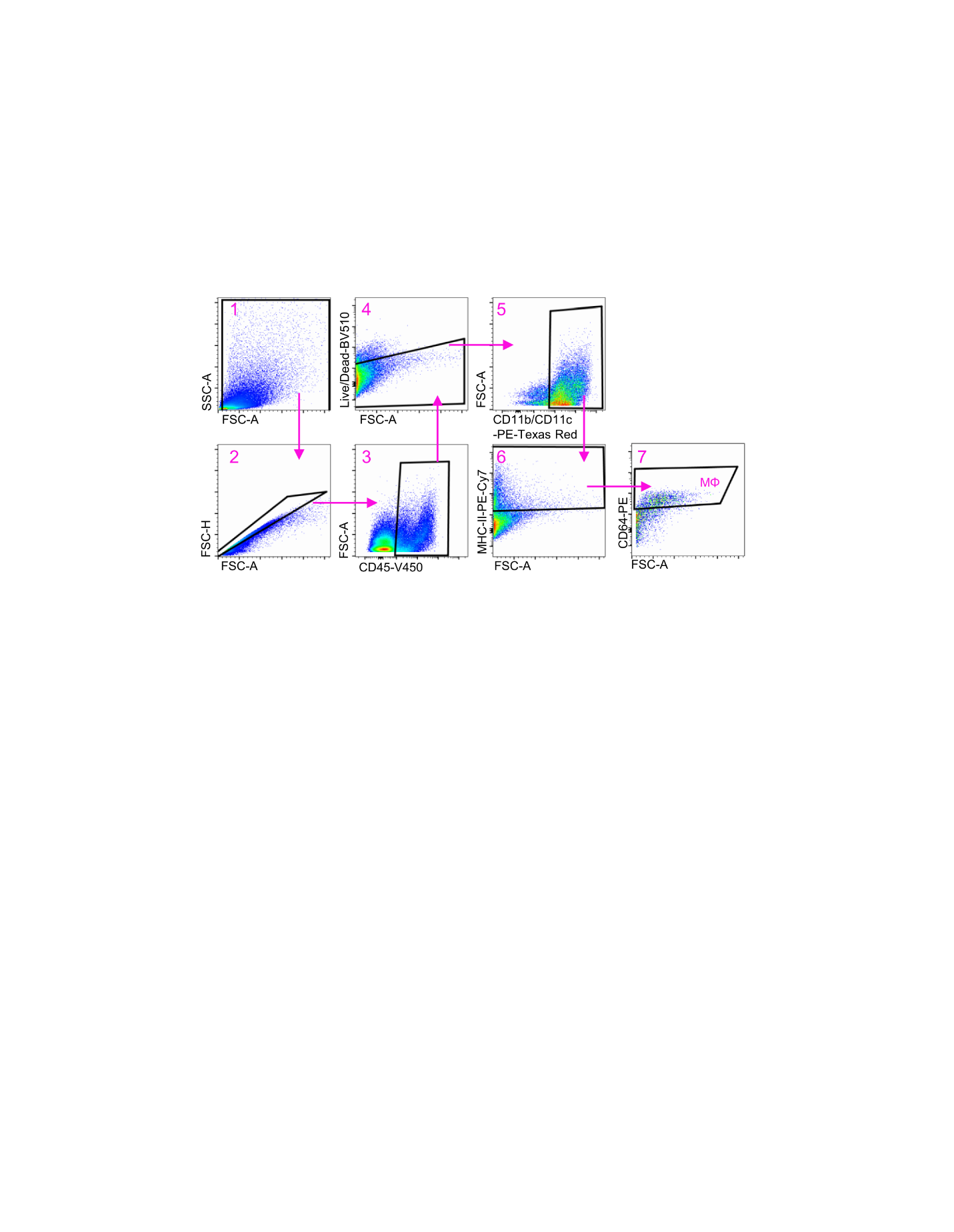
Supplemental Figure 6. Gating strategy for M1/M2 polarization in BALF.** BALF was harvested on day 3 after thoracic irradiation, and single-cell suspensions of BALF were stained with anti-CD45, anti-CD11b, anti-CD11c, anti-MHC-II, anti-CD64, anti-CD86, anti-CD206, and a live/dead staining kit. The expression of CD86 and CD206 were gated by CD45^+^CD11b^+^CD11c^+^MHC-II^+^CD64^+^ cells, which were defined as macrophages (MФ) in the BALF.


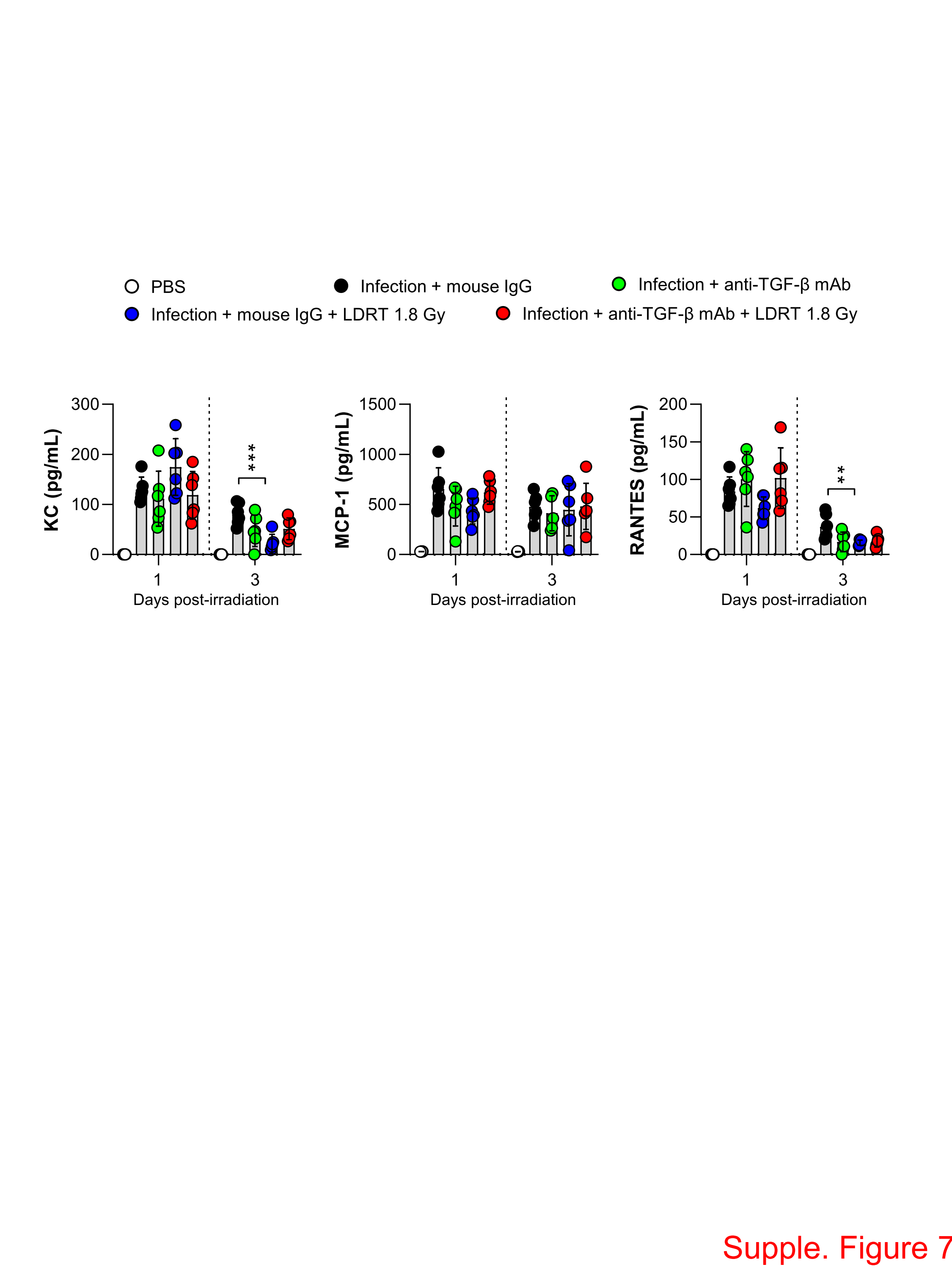


**Supplemental Figure 7. TGF-β blocks the expression of chemokines via LDRT-induced chemokine expression.** Mice were intraperitoneally administered anti-TGF-β monoclonal antibody (200 μg/mouse) or mouse IgG antibody (isotype control) 4 h prior to thoracic irradiation (1.8 Gy). BALF was harvested on days 1 and 3. The levels of chemokines (KC, MCP-1, and RANTES) in the supernatant of BALF were analyzed using the CBA assay. Data are presented as the mean ± SD (n = 6 mice/group). Statistical analysis was performed using two-way ANOVA in conjunction with Tukey’s test. ^**^*p* < 0.01 and ^***^*p* < 0.001.
